# Study of Early-Age Hydration, Mechanical Properties Development, and Microstructure Evolution of Manufactured Sand Concrete Mixed with Granite Stone Powder

**DOI:** 10.3390/ma16134857

**Published:** 2023-07-06

**Authors:** Jianghua Wang, Cuizhen Xue, Yu Zhang, Qiangming Li, Yixuan Han, Hongxia Qiao

**Affiliations:** 1 College of Civil Engineering, Lanzhou University of Technology, Lanzhou 730050, China; wangjh049@163.com (J.W.);; 2 Western Advanced Civil Engineering Materials Innovation Research Center, Lanzhou 730050, China; 3 Jiangsu Provincial Highway Business Development Center, Nanjing 210004, China; 4 JSTI GROUP, Nanjing 211112, China; 5 State Key Laboratory of Safety and Health of In-Service Long Span Bridges, Nanjing 211112, China

**Keywords:** granite stone powder, manufactured sand concrete, hydration products, pore structure, interfacial transition zone

## Abstract

This study explored the potential of granite stone powder (GSP) as a supplementary cementitious material (SCM). The 72 h early hydration process stages of GSP-mixed slurry were analyzed in depth, and the mechanical properties of manufactured sand concrete (MSC) mixed with GSP were investigated. Physical phase types, morphological characteristics, and pore structure evolution were investigated using an X-ray diffractometer, scanning electron microscope, and mercury intrusion approach (MIP). Atomic force microscopy was used to show the interface transition zone between aggregate and slurry in phase images, height images, and 3D images, allowing quantification of ITZ and slurry by calculating the roughness. Gray entropy analysis was used to evaluate the significance of the effect of pore size distribution parameters on mechanical strength, and the GSP-content-mechanical-strength gray model GM (1, 1) was established to predict mechanical strength. The results indicate that, compared with the reference group, the GSP cement slurry system exhibited a delayed hydration process acceleration rate, with a 1.04% increase in cumulative heat of hydration observed in the 5% test group and an 11.05% decrease in the 15% test group. Incorporating GSP in MSC led to decreased mechanical properties at all ages, with significant decay observed when incorporation ranged from 10% to 15%. Although the type of hydration products remained unchanged, there was a decrease in the number of C-S-H gels and gel pores, while large pores increased, resulting in increased porosity and roughness of the interface transition zone and slurry. Large pores (>1000 nm) were found to have the greatest influence on mechanical strength, with gray correlation above 0.86. The GM (1, 1) model yielded accurate predictions, showing good agreement with measured data and thus it can be identified as belonging to a high-precision prediction model category. These findings provide theoretical support and a reference for applying GSP as an SCM, laying the groundwork for data-based specification development.

## 1. Introduction

In recent years, the rapid development of China’s infrastructure, with the annual consumption of concrete at about 1.8 billion cubic meters, has resulted in a serious decline in high-quality natural sand reserves. In some areas, natural sand resources are nearly depleted and cannot meet the growing demand for engineering construction. The gap between supply and demand is increasing. However, the government and the public have started focusing on the ecological protection of the environment and, with the continuous introduction of environmental policies promoting the environmental transformation of the sand and gravel aggregate industry, the amount of mechanism sand is now beginning to increase. Another issue is that before use, sand needs to go through several processes, such as crushing and screening, which inevitably produces a large amount of stone powder. In recent years, the annual production of mechanism sand in China has become as high as 20 billion tons, resulting in the discharge of between approximately 600 million and 1.2 billion tons of stone powder per year [1]. A large amount of stone powder, if not properly used, not only causes serious pollution in the mining environment (e.g., stone powder infiltrates into rivers, changes the pH of agricultural soil, and causes landslides), but also results in higher disposal costs, which is a waste of resources. Therefore, there is an urgent need to seek new, green methods for the reuse of stone powder [2].

The uneven distribution of common SCM resources such as fly ash and slag has led to an increasing conflict between SCM supply and demand. Therefore, there is an urgent need to develop a new SCM. If stone powder can replace part of the cement as an SCM, this can not only turn stone powder into a resource but also reduce building energy consumption and environmental pollution [3,4]. Scholars at home and abroad have carried out research on stone powder as SCM. The available research results show that limestone powder can be used as a new type of SCM. In concrete, the mechanism of limestone powder is divided into four types: the crystalline nucleation effect [5], the filling effect [6], the dilution effect [7], and the chemical effect [8]. Based on this research, standards have been developed in Europe [5,9], Canada [10,11], the United States [12], and China [13,14].

Currently, domestic and foreign scholars are actively exploring the potential of granite powder as an SCM and its effect on concrete performance and mechanism in concrete. Li et al. [15] used granite powder (GSP, i.e., particles less than 0.075 mm in machined sand) to replace fly ash as an SCM. The results showed that granite powder could enhance the work performance of MSC and that the late compressive strength, flexural strength, and modulus of elasticity of MSC increased when 20% of the MSC was replaced by granite powder. Elmoaty, Kumar, Rashwan, Sadek et al. [16,17,18,19] used different percentages of granite powder (granite cutting waste, GCW) in equal amounts to replace cement in order to study its effect on the properties of MSC. The results showed that the equivalent substitution of different percentages (5–10%) of GCW in cement had favorable effects on the mechanical properties of concrete [17]. Furthermore, the mechanical strength values of the prepared river sand concrete met the standard requirements and, when the GCW substitution was 20%, the mechanical properties of the MSC were not adversely affected [18]. The test results showed that the compressive strength of self-compacting concrete increased by 7.8%, 23.1%, and 39.3% at 30%, 40%, and 50% of GCW substitution, respectively. To further investigate the mechanism of granite powder as an SCM on the mechanical properties of concrete, scholars carried out microscopic experiments [19]. Zhang, Shen et al. [20,21] showed that the performance of GSP-doped ultra-high-performance concrete on a macroscopic scale is closely related to the pore structure on the microscopic scale. As GSP can fill the excess voids and reduce the pores in cement paste, this improves its denseness, reduces water secretion in the interface transition zone, and improves its durability and volume stability [21].

Jain, Xue, Liu et al. [22,23,24] showed, via SEM, EDS, XRD, and FTIR analysis, that the GSP is mostly inert at the microscopic level and only limited chemical activity exists in trace amounts, which accelerates the early cement hydration rate, thus accelerating the development of early concrete strength. GSP mainly plays a physical filling role in concrete, and the appropriate amount of GSP can have a good filling effect and reduce concrete porosity, improving the mechanical properties of concrete. However, the dilution effect of the stone powder and the formation of interfacial defects may have some adverse effects on the mechanical properties of concrete when the addition of stone powder exceeds a certain critical value. This is because the less active stone powder has only a limited impact on the chemical reaction, resulting in a reduction in the generation of hydration products due to the reduction in the total amount of cementitious material when an excess substitution of stone powder is used [25].

In summary, granite powder mainly has crystalline nucleation, filling, chemical, and dilution effects in concrete, improving its macroscopic mechanical properties [17]. However, there are still some deficiencies in the research [26,27] and most of the previous studies were carried out on GCW as an SCM, with river sand concrete as the research vehicle, while mechanism sand is the future development trend and the mechanism of producing sand inevitably produces by-products (stone powder). In addition, the research on the effect and mechanism of GSP on the mechanical properties of MSC is not systematic enough at this stage and no systematic conclusion has been formed. In view of this, this paper carries out research on the influence of GSP dosing on the macro- and micro-mechanical properties of MSC, calculates the gray correlation between mechanical strength and pore size distribution parameters based on the gray entropy theory, and establishes the gray model GM (1, 1) of GSP dosing–mechanical strength at different ages. The findings of the study lay a data basis for the promotion and application of GSP and the development of relevant specifications.

## 2. Raw Materials and Test Methods

### 2.1. Raw Materials

The following raw materials were used for the test:

(1) Cement: P·O 42.5 grade ordinary silicate cement produced by Qilianshan Cement Factory.
(2) Fly ash: Secondary fly ash produced by Jingyuan Power Plant in Baiyin City, Gansu Province.
(3) Granite stone powder: Powder obtained by granite mechanism sand through a 0.075 mm sieve.
(4) Granite mechanism sand: Sand with a fineness modulus of 3.5 produced by Bashi Gou quarry in Yuzhong County, Lanzhou City, with an apparent density of 2643 kg/m^3^, a tight-packing density of 1657 kg/m^3^, a loose-packing density of 1476 kg/m^3^, and a crushing value of 18.18%.
(5) Granite mechanism stone: Stone in two particle size ranges, 5~10 mm and 10~20 mm, with mass ratio of 4:6, respectively; the apparent density is 2696 kg/m^3^, and the porosity is 43.3%.
(6) Water: Lanzhou city tap water.
(7) The water reducer: High performance polycarboxylic acid water reducer (PCA), water-reducing rate 43%.

As can be seen from Table 1, GSP and cement have a similar chemical composition but different contents. The content of SiO_2_ and Al_2_O_3_ of GSP is about twice that of cement, while SiO_2_ and Al_2_O_3_ react with Ca (OH)_2_ to form hydrated calcium silicate and calcium aluminate, indicating that GSP has some potential to be used a SCM.

**Figure 1:**
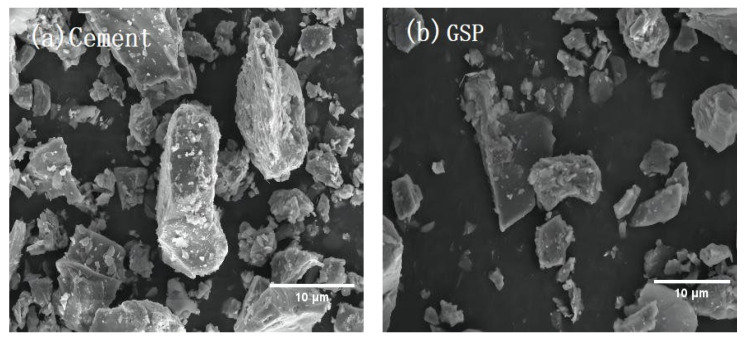
SEM photos of cement and GSP (×5000).

Figure 1 displays the SEM images of cement and GSP, showing that the microscopic morphology of cement particles is significantly different from that of GSP. The cement is overall denser, with more flocs attached to the particle surface, and some of the particles are close to elliptical in shape. In contrast, the overall structure of GSP is loose; the outline of particles is clear, with obvious irregular angles, and the particles are mainly fine and flaky and in the form of flakes and flat plates.

**Figure 2:**
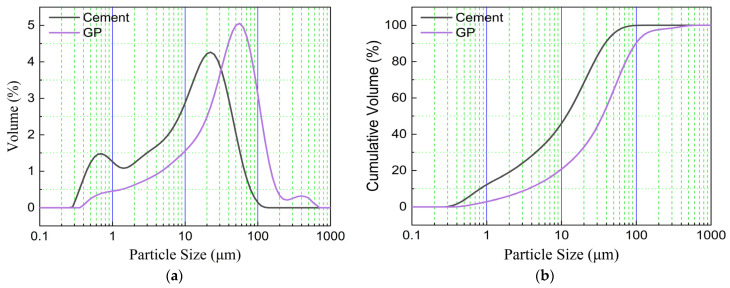
Cement and GSP laser particle size curve. (**a**) distribution curves; (**b**) accumulation curve.

Figure 2 and Table 2 show the laser particle size curves and the particle size distributions for cement and GSP, respectively. From Figure 2, it can be seen that the laser particle size distribution curve and the cumulative curve of GSP are shifted to the right compared with that of cement, which indicates that the particle size is coarse overall. The strength of the crystalline nucleation of stone powder is related to the particle size of stone powder: the larger the particle size, the smaller the crystalline nucleation, and the weaker the effect of accelerating the hydration process.

### 2.2. Experimental Program

In this study, concrete with a strength grade of C30 was used to investigate the effect of a GSP admixture (0%, 5%, 10%, 15%) on the mechanical properties (compressive strength, splitting tensile strength, flexural strength, etc.) and the evolution of the heat of hydration of cement–GSP composite cementitious materials. The specific test protocol is presented in Table 3. Microstructural analysis techniques such as X-ray diffractometer (XRD), scanning electron microscope (SEM), mercury piezometer (MIP), and atomic force microscope (AFM) were used to analyze the mechanism of the effect of GSP admixture on the hydration products, microstructural characteristics, pore structure, and critical surface transition zone of the concrete. The mixture proportions of concrete are shown in Table 3, designed according to the proportional design method specified in JGJ 55-2011 “Proportional design procedures for ordinary concrete”. The specific test protocol is shown in Table 4 and Table 5.

### 2.3. Test Methods

#### 2.3.1. Laser Particle Size Test

Through laser particle size test, the particle size distribution characteristics, average particle size, surface area, and other physical parameters of cement and granite powder can be characterized, which lays a foundation for evaluating the potential activity of granite powder as a supplementary cementitious material instead of cement and provides a theoretical basis for designing the replacement amount of powder in concrete mix proportioning with added granite powder. A Mastersizer 2000 laser particle size analyzer was used to test the particle size distribution of cement and granite stone powder. The test method was wet, and the materials mentioned above were sonicated in dispersant (anhydrous ethanol) for 2 min before the test.

#### 2.3.2. Heat of Hydration Test

Each stage of the 72 h early hydration process (preinduction, induction, acceleration, deceleration, and stability) of GSP incorporation was deeply analyzed to obtain the action mechanism of GSP in each stage. This test was carried out by means of an 8-channel TAM Air isothermal calorimeter based on ASTM C1679 [28]. According to Table 3, 100 g of the composite gelling material and 47 g of deionized water were stirred in a blender at a high speed for 60 s and then about 10 g of the slurry was weighed into an ampoule. The temperature of the apparatus was controlled at 25.00 ± 0.02 °C, and the measurement duration was 72 h. The rate of heat of hydration as well as the cumulative heat of hydration of the samples were recorded. For each sample, two sets of tests were performed and the average of the two results was taken as the test result.

#### 2.3.3. Concrete Mechanical Properties Test

The mechanical properties of concrete are an important parameter for evaluating the feasibility and rationality of structural design. In order to study the influence of GSP on the concrete mechanical properties, mechanical property tests were carried out. The concrete mechanical properties test was conducted in accordance with GB/T 50081-2019: standard for test methods of concrete physical and mechanical properties [29]. The size of compressive strength and tensile strength specimens was 100 mm × 100 mm × 100 mm; the size of flexural strength specimens was 100 mm × 100 mm × 400 mm; the standard number of days for which the concrete specimens were cured were 3 d, 7 d, and 28 d; each group had 3 parallel specimens; and the arithmetic mean of the test values of the 3 parallel specimens was used as the strength value of each group of specimens.

#### 2.3.4. X-ray Diffraction Test (XRD)

In order to characterize the effects of age and granite powder content on concrete phases through XRD testing, before X-ray powder diffraction was performed, the concrete samples were soaked in ethanol for 3 days to stop hydration. Then, the slurry was taken, ground into a fine powder sample, and passed through a 75 mm sieve. A Bruker D8 advance model X-ray diffractometer was used for the physical phase examination of the samples to be tested. The parameters were set as follows: scanning range 10° to 80°, step size 0.02°, tube voltage 20 kV, tube current 2 mA, and scanning speed 8°/min.

#### 2.3.5. Scanning Electron Microscopy (SEM)

In order to investigate the effects of GSP content on the types, quantities, and morphology of hydration products in MSC through scanning electron microscopy (SEM), a Hitachi HITACHI SU8010 (Beijing, China) scanning electron microscope was used to study the effect of GSP doping on the microstructural characteristics of MSC. Using a large cutting machine, samples with side lengths of up to 10 mm were intercepted from the fracture surface of split tensile specimens. Then, the samples were soaked in anhydrous ethanol for 3 days to stop hydration and dried under vacuum at 50 °C for 24 h. Finally, the surface of the samples was sprayed with gold before SEM testing.

#### 2.3.6. Mercury Intrusion Porosimetry Test (MIP)

In order to study the influence of different dosages of GSP on the pore structure of MSC through MIP analysis, the porosity and pore size distribution of test groups with different doses of GSP (0%, 5%, 15%) were investigated after curing for 28 d using MIP. Firstly, a wire cutter was used to cut the 100 mm × 100 mm × 100 mm cubic concrete specimens along the vertical direction into 10 mm × 10 mm × 100 mm rectangular mounted specimens. Then, from the center of the rectangular specimen block, 10 mm × 10 mm × 10 mm concrete specimens were taken and dried in an oven at 50 °C for 24 h. Next, a mercury intrusion porosity test was performed on an AutoPore v9620 model mercury-pressure instrument with maximum and minimum test pressures of 400 MPa and 0.005 MPa, respectively; a mercury contact angle of 130°; and a measurable pore size (3.6~500,000 nm) of hardened concrete.

#### 2.3.7. Atomic Force Microscopy Test (AFM)

In order to study the effect of GSP on the interface transition zone (ITZ) of MSC, including phase diagrams, height maps, and 3D terrain images, atomic force microscopy (AFM) was used. The corresponding AFM software was used to quantitatively analyze the roughness of the ITZ and the paste, and to evaluate the degree of influence of granite powder. An atomic force microscope of the Innova type from Bruker, with a probe of the RETSP type, Si as the material, a micro-cantilever elastic modulus of 40 N-m^−1^, a vibration frequency of 300 kHz, scanning set in tap mode, a scanning resolution of 256 × 256, and a scanning frequency of 0.5 Hz was used. The interfacial transition zone was identified by using the optical lens with the help of AFM, and a 30 μm × 30 μm image of the scanned area was obtained. The specimens were prepared as follows: firstly, 100 mm × 100 mm × 100 mm cubic concrete specimens with a curing age of up to 28 d were cut into 20 mm × 20 mm × 100 mm rectangular specimens along the vertical direction using a large cutting machine. Then, 20 mm × 20 mm × 2 mm sheet specimens were taken from the center of the rectangular specimens using a wire cutting machine. These specimens were cut with 600-mesh, 800-mesh, 1000-mesh, 1500-mesh, and 100-mesh specimens in order. After the specimens were repeatedly ground with 600-mesh, 800-mesh, 1000-mesh, 1500-mesh, and 2000-mesh sandpaper, they were polished with 1 μm and 0.25 μm diamond polishing solutions. Finally, the specimens were placed in an ultrasonic cleaner and cleaned with anhydrous ethanol for 5 min to remove any powder from the concrete surface and the pores. To complete the specimen preparation, the specimens were put into sealed bags for storage.

## 3. Results and Discussion

### 3.1. Evolution of the Heat of Hydration of the Cement–GSP Composite Cementitious Material System

**Figure 3:**
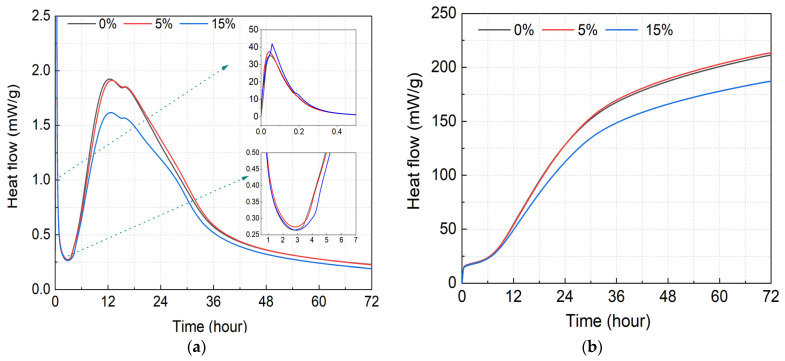
Evolution of the heat of hydration of the cement–GSP composite cementitious material system. (**a**) Heat of hydration rate; (**b**) Cumulative heat of hydration.

Figure 3a shows the heat of hydration rate of a cement–GSP composite cementitious material system. It can be seen that as the GSP admixture increases, the overall heat ofhydration rate decreases. In addition, the heat of hydration rate curves of different GSP admixture cementitious material systems are relatively similar. Similar to the hydration process of cement slurry, the hydration process of the cement–GSP gelling material system is also divided into five stages: pre-induction, induction, acceleration, deceleration, and stabilization [30,31]. When the cementitious material is added to water, the first exothermic peak, caused by the formation of calcium alumina, is formed rapidly and this exothermic peak shows that GSP accelerated the rate of the pre-induction phase of cement hydration, and the incorporation of a larger amount of GSP delayed the pre-induction phase [32]. When the cement–GSP composite cementitious material system enters the acceleration period, its heat of hydration rate slows down with the increase in the GSP admixture, and the difference in the heat of hydration rates of different GSP admixture test groups gradually increases with time. Compared with the 0% test group, the maximum heat of hydration rates of the 5% and 15% test groups decreased by 0.63% and 15.96%, respectively, indicating that the 5% GSP dosing has little effect on the heat of hydration rate of the cementitious material system, which is related to the crystalline nucleation of GSP, which accelerates the hydration process of cement [33]. Meanwhile, the dilution effect of excessive GSP begins to slow down the hydration process of the cement. Due to reduced cement quantity, the promotion effect does not compensate for this slowing down. With the coverage of a large number of hydration products, the hydration reaction rate reduces and enters the deceleration period [34]; gradually, the diffusion rate begins to control the hydration process [35]. Specifically, at about 22–33 h, the heat of hydration rate of the 5% test group is slightly greater than that of the 0% test group, indicating that the diffusion rate of the GSP-doped cementitious material system is greater than that of the 0% test group during the deceleration period, but the deeper reasons for this need to be explored.

Figure 3b shows the cumulative heat of hydration of the cement–GSP composite gelling material system, which decreases with the increase in the GSP admixture. Compared with the 0% test group, the cumulative heat of hydration of the 5% test group increases by 1.04% and that of the 15% test group decreases by 11.50%, indicating that the hydration process of the 0% test group is basically equivalent to that of the 5% test group. However, this does not mean that the corresponding macroscopic mechanical properties are basically the same, because the mechanical properties are related to the hydration process of the cementitious material system as well as the type of internal hydration products, the microstructure, the pore structure, the interface transition zone, and other factors [36,37,38]. Therefore, further research is necessary.

### 3.2. MSC Mechanical Properties Test

**Figure 4:**
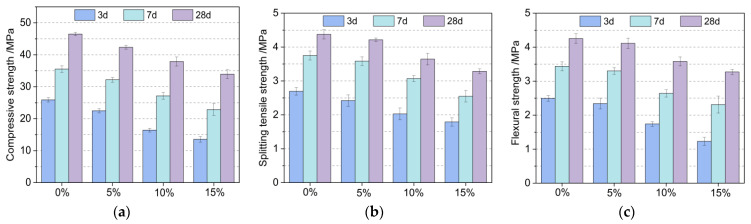
Mechanical properties test results of MSC mixed with GSP. (**a**) Compressive strength; (**b**) Splitting tensile strength; (**c**) Flexural strength.

As can be seen from Figure 4, the compressive strength, the splitting tensile strength, and the flexural strength of the machine-made sand concrete showed the same change pattern with the increase in the GSP admixture, i.e., the strength of the specimens gradually decreased with the increase in the GSP admixture. Compared with the 0% test group, the 28-d compressive strength, splitting tensile strength, and flexural strength of the 5% test group decreased by 8.9%, 4.6%, and 3.1%, respectively; the 28-d compressive strength, splitting tensile strength, and flexural strength of the 10% test group decreased by 18.5%, 16.9%, and 15.8%, respectively; and the 28-d compressive strength, splitting tensile strength, and flexural strength of the 15% test group decreased by 26.9%, 25.8%, and 23.1%, respectively. This is because the activity of GSP is much smaller than the reactive activity of cement and after the addition of GSP, the content of cement decreases, the hydration products per unit volume of cementitious material decrease, and the dilution effect increases [38], leading to a decrease in the mechanical properties of concrete with the increase in the stone powder admixture.

Figure 4 shows that as the curing age increased, there was a gradual increase in the compressive strength, the splitting tensile strength, and the flexural strength of the same GSP-doped specimens. In addition, the reduction in strength was smaller in the early stage than in the later stage. In the early stage of maintenance, the 7-d compressive strength increased by 37.14%, 43.21%, 65.25%, and 68.63%; the 7-d splitting tensile strength increased by 39.41%, 47.93%, 51.23%, and 42.46%; and the 7-d flexural strength increased by 38.15%, 41.03%, 51.72%, and 87.80% compared with the 3-d strengths, respectively. The incorporation of stone powder had a more significant effect on the pre-strength promotion of the machine-made sand concrete, due to its crystalline nucleation effect, which reduces the nucleation barriers and provides more nucleation matrices for the growth of hydration products [39], accelerating the early strength growth of GSP-doped MSC.

In summary, as a supplementary cementitious material, GSP has both an unfavorable side (that is, a dilution effect, and the greater the amount of admixture, the stronger the dilution effect, and the fewer the number of hydration products of the cementitious material system), and a favorable side (that is, in the pre-conservation period, GSP in the cementitious material has a crystal nucleation effect, which accelerates the hydration of the cement). When the amount of the stone powder admixture is large, the unfavorable effect of the stone powder is more obvious, which is macroscopically shown as a decrease in the mechanical strength with an increase in the GSP admixture. Combined with the thesis test results, in order to ensure the strength of the concrete, the GSP admixture should be controlled to a value within 5%. In addition, to improve the activity of GSP in practical application, the powder can be further ground to a finer state to increase the nucleation sites of hydration products and improve its crystalline nucleation effect.

### 3.3. Phase Analysis of the Cement–GSP Composite Cementitious Material System

**Figure 5:**
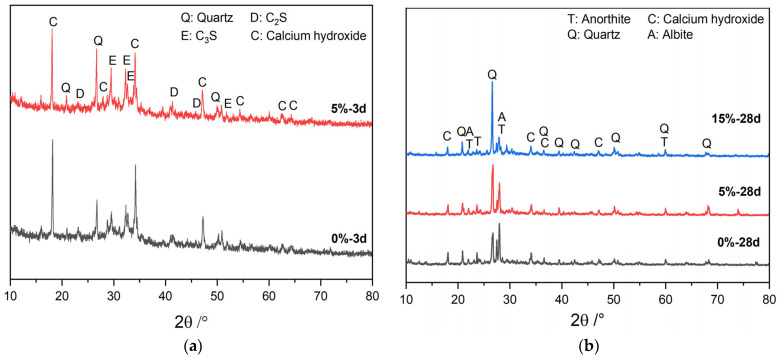
XRD diagrams of different GSP doping gelling material systems at the same age: (**a**) 3 d; (**b**) 28 d.

Figure 5 shows that the overall characteristics of the XRD patterns of the GSP-doped specimens are basically the same as those of the 0% test group at the same age, and no new phases were found, indicating that no new hydration products were generated in the cementitious material system when GSP replaced cement. In Figure 5a, the peak value of calcium hydroxide is only slightly lower in the 5% test group than in the 0% test group, possibly due to the crystalline nucleation effect of GSP, which promotes the crystallization of calcium hydroxide at the early stage of hydration and compensates to some extent for the reduction in hydration products caused by the decreased cement quantity. In Figure 5b, the calcium hydroxide diffraction peak decreases as the age of maintenance increases, and the C_3_S and C_2_S diffraction peaks almost disappear. Combined with the aforementioned heat of hydration test, this shows that the secondary hydration of calcium hydroxide and fly ash occurred and the degree of hydration was deepening [40]. In addition, with the increase in GSP doping, the peak of quartz (of which the main component is silica) gradually increases, indicating that most of the silica mineral components in GSP belong to inactive substances and the dilution effect of GSP leads to a decrease in the hydration degree [41], which also decreases calcium hydroxide generation, hence being the reason why the peak in calcium hydroxide is lower in the 15% test group than in the 0% test group in Figure 5b.

### 3.4. SEM Analysis of MSC with Different GSP Doping Levels

**Figure 6:**
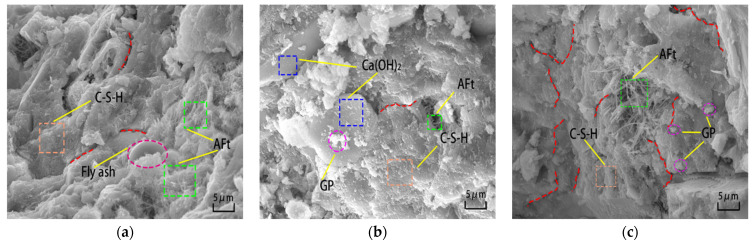
SEM images of different GSP test groups (magnification 2000 times). (**a**) 0% test group; (**b**) 5% test group; (**c**) 15% test group.

From Figure 6, it can be seen that the MSC microstructure gradually deteriorates with the increase in GSP doping. Figure 6a shows the SEM image of the 0% test group. In the image, the “worm-like” C-S-H gel is greater in quantity [42], grows together, and intertwines with other hydration products to form a dense mesh structure without large gaps in the structure, making the overall microstructure relatively dense except for a few unhydrated spherical fly ash particles. Figure 6b shows the SEM image of the 5% test group with no obvious unhydrated cement and fly ash particles, indicating that GSP played a role in nucleation, accelerated the hydration process of the cementitious material, and provided nucleation sites for the growth of C-S-H [43]. In addition, a small number of GSP particles filled the gaps between the needle- and rod-like calcium alumina [44]. However, compared with the 0% test group, the surface denseness of its hydration products, such as C-S-H gel, was poor, the bonding between the matrix was looser, and there were a few fine cracks and folds on the surface of the hydration products, and these factors led to the degradation of the macroscopic mechanical properties of MSC to some extent. Figure 6c presents the SEM image of the 15% test group, showing that when the GSP doping increases, the number of cracks and the number of free GSP particles inside the structure increase and the overall structure is looser, with more weak links. This is because part of the GSP exists in the free form at the cement stone or interface, weakening the bond between mortar and aggregate, and the number of C-S-H gels produced by hydration due to the dilution effect of GSP in excess cannot fill the gap between calcium alumina crystals [45], which is not conducive to strengthening the concrete, and the macroscopic expression of MSC mechanical strength further decreases.

### 3.5. Effect of GSP Doping on the Evolution Pattern of the MSC Pore Structure

**Figure 7:**
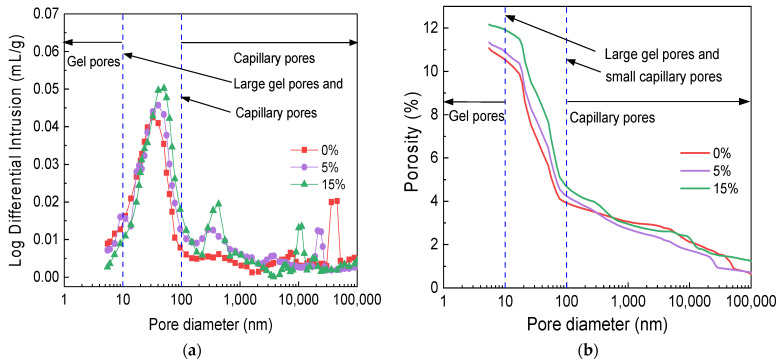
MIP test results of different GSP test groups. (**a**) Differential curve of pore size distribution; (**b**) Cumulative pore size distribution curve.

Figure 7 shows the MIP test results for different GSP test groups. In Figure 7a, which shows the differential curve of pore size distribution, with an increase in stone powder doping, the most available pore size (the log differential intrusion peak in Figure 7a) increased by 24.26% and 55.43% in the 5% and 15% test groups, respectively, compared with the 0% test group. Figure 7b shows the variation in cumulative porosity with pore size, which is 11.08%, 11.34%, and 12.16% for the 0%, 5%, and 15% groups, respectively, further indicating that GSP incorporation reduces MSC density.

Table 6 shows the pore size distribution of MIP in different GSP test groups. To better study the effect of stone powder on the pore size distribution, the pores were divided into four types: gel pores, with a pore size less than 10 nm; transition pores, with a pore size between 10 and 100 nm; capillary pores, with a pore size between 100 and 1000 nm; and macropores, with a pore size greater than 1000 nm [46]. With the increase in GSP doping, the number of MSC gel pores and macropores decreased, the number of capillary pores increased, and the number of transition pores remained basically the same. Compared with the 0% test group, the number of gel pores decreased by 15.15% and 56.45%, and the number of macropores decreased by 13.47% and 12.38% when the GSP doping was 5% and 15%, respectively. This is because the weaker GSP replaces cement, reducing the number of cement particles in the cementitious material. This in turn reduces the number of C-S-H gels generated by hydration, decreasing the number of MIP gel pores, and the larger the GSP dosing, the greater the reduction in the number of gel pores. GSP mainly plays the role of a micro-aggregate filling. GSP particles fill some of the pores of >1000 nm, but the GSP filling effect is limited and the vast majority of GSP particles remain in the free state. However, relative to the 0% test group, in the other groups, the number of large pores decreases.

In conclusion, the incorporation of GSP instead of cement degrades the pore structure of MSC and when the amount of GSP increases, the degree of deterioration of the pore structure increases.

### 3.6. AFM Analysis of MSC with Different Levels of GSP Doping

**Figure 8:**
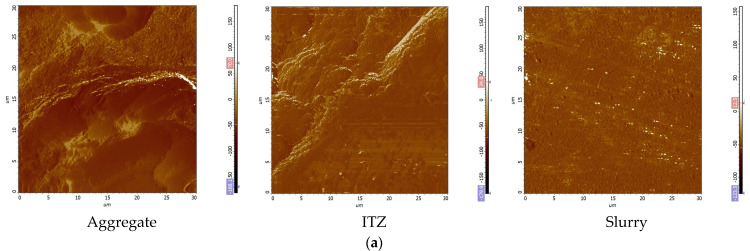

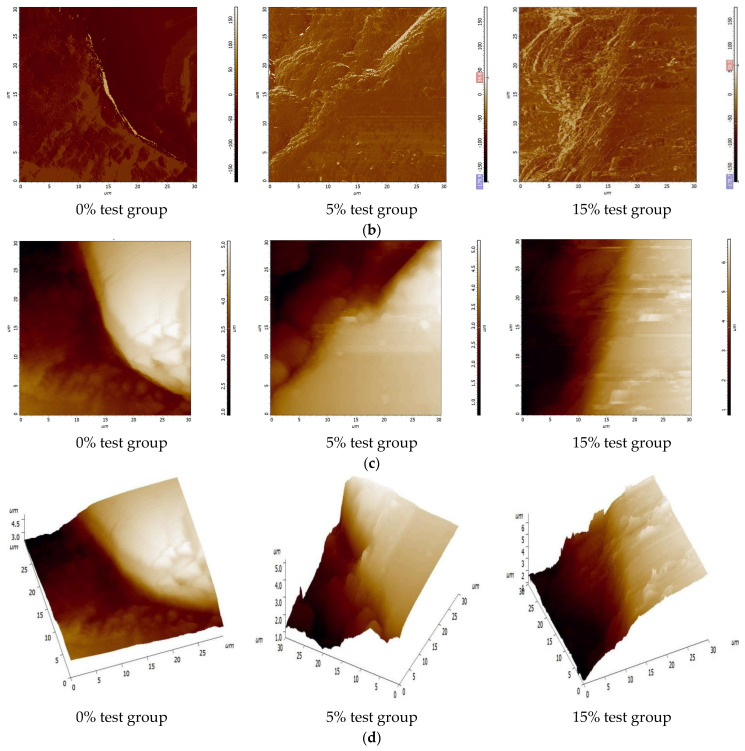
AFM scan images of different GSP test groups. (**a**) Probe scanning direction (Aggregate→ITZ→Slurry); (**b**) ITZ phase images; (**c**) ITZ height images; (**d**) ITZ 3D images.

Figure 8a shows the probe scanning direction, from the aggregate to the ITZ to the slurry transition, so as to obtain a 30 μm × 30 μm ITZ scanning area image (phase diagram, height diagram, 3D stereogram). Figure 8b shows the ITZ phase diagram. As per Figure 8b, it can be seen that the number of ITZ gullies in the MSC of the 0% test group is low, the surface in the region has no obvious undulations, and the structure is relatively flat, while with the increase in GSP doping, the number of ITZ gullies starts to increase and the flatness of the region decreases, and when the doping amount is 15%, the number of ITZ gullies increases significantly and spreads over almost the whole region, and the structure presents a more loose state [47]. It shows that the ITZ is the densest in the 0% test group, while the structure is the loosest in the 15% test group, and this conclusion is consistent with the SEM observations. This is because, compared to the cement, GSP, which replaces cement, has weaker activity and decreases the hydration products of cementitious materials. In addition, the free state of stone powder affects the bond between aggregate and cement stone, resulting in weaker adhesion between the matrix, sparse structure formation inside the concrete, and an increase in porosity, leading to a decrease in macroscopic mechanical properties [48].

**Figure 9:**
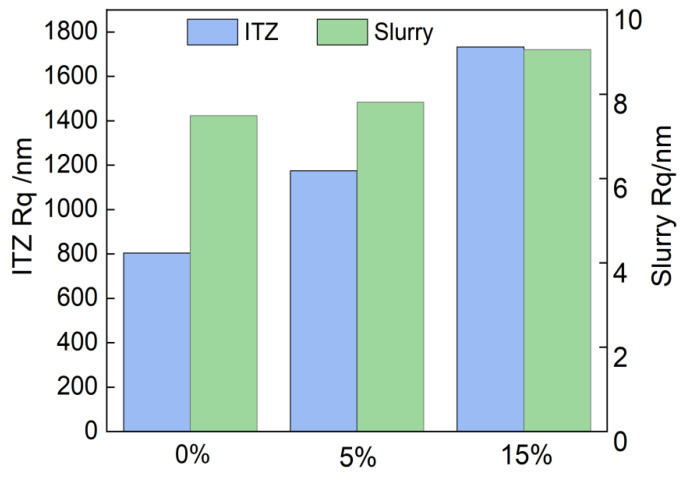
ITZ and slurry roughness for different GSP test groups.

Figure 9 shows the roughness (Rq) of the ITZ and the slurry for different GSP test groups. It can be seen that with the increase in the GSP admixture, the Rq of the ITZ and the slurry increases, i.e., the structure tends to be uneven and in a loose state, and the denseness decreases. Relative to the slurry, the ITZ has a larger Rq, mainly due to the loose and porous microstructure characteristics of the ITZ. Compared with the 0% test group, in the 5% and 15% test groups, the Rq of the ITZ increased by 45.84% and 115.28%, respectively, while the Rq of the slurry increased by only 7.81% and 20.93%, respectively. It shows that the 0% test group shows a sufficient growth in internal hydration products, fewer internal defects, and a denser slurry structure, while the incorporation of GSP has a deterioration effect on the internal structure, and the ITZ is affected to a greater extent than the slurry. Relatively speaking, the ITZ is a weak link in the concrete: the greater the amount of GSP incorporation, the less dense the structure becomes, and its deterioration phenomenon is more serious. When subjected to external loading damage, the ITZ begins to develop and expand, leading to the accelerated destruction of the mechanism of sand concrete, having a considerable impact on its mechanical strength. Macroscopically, GSP has a negative impact on the mechanical properties of MSC [38].

## 4. Gray Entropy Analysis and GM (1, 1) Mechanical Strength Prediction Modeling

### 4.1. Gray Entropy Analysis of Mechanical Strength and Pore Size Distribution Parameters

“Gray entropy analysis” is a systematic method used for analyzing random factors under “small sample and poor information” conditions, aiming to find correlations in the processed data sequence. In order to quantitatively analyze the significance of pore size distribution parameters on mechanical strength, the 28-day compressive strength, splitting tensile strength, and flexural strength were used as the parent series, while gel pore, transition pore, capillary pore, and large pore were used as the sub-series representing the pore size distribution parameters. The values of the influence of pore size distribution parameters on mechanical strength were calculated according to the formula presented in the literature [49], and the gray entropy correlation between mechanical strength and pore size distribution parameters was obtained. These results are shown in Table 7.

Table 7 shows that, except for capillary pores with a pore size distribution between 100 and 1000 nm, the gray correlation between MSC mechanical strength and the other pore size distribution parameters is high. The gray correlation between compressive strength and pore size distribution parameters, in descending order, was >1000 nm (large pores), <10 nm (gel pores), 10–100 nm (transition pores), and 100–1000 nm (capillary pores). Similarly, the gray entropy correlation between flexural and splitting tensile strengths and pore size distribution parameters, in descending order, was >1000 nm (large pores), 10–100 nm (transition pores), <10 nm (gel pores), and 100–1000 nm (capillary pores). These results indicate that macropores have the greatest influence on the mechanical properties of MSC, followed by gel pores and transition pores.

### 4.2. Development of GM (1, 1) Prediction Model for the Mechanical Strength of GSP-Doped MSC

Gray system theory has been recognized as an effective method for dealing with discrete data and uncertainty arising from incomplete information. By establishing a GM (1, 1) gray model based on age-specific GSP doping–mechanical strength data, it becomes possible to predict the mechanical strength of MSC at certain ages, enabling the correlation between GSP doping and the macroscopic mechanical strength of MSC to be determined. This, in turn, will facilitate regulating GSP doping to optimize the mechanical properties of MSC.

#### 4.2.1. Model Building

The specific calculation procedures for the model are described in literature [50].

Generalized compressive strength model for MSC with different levels of GSP doping at various ages.

$$3\;d:\;{\hat{X}}^{\left(1\right)}(c)=-96.06e^{-0.052c}+121.96;$$

$$7\;d:\;{\hat{X}}^{\left(1\right)}(c)=-204.73e^{-0.034c}+240.25;$$

$$28\;d:\;{\hat{X}}^{\left(1\right)}(c)=-404.77e^{-0.022c}+451.24.$$

Generalized model for the splitting tensile strength of MSC with different GSP doses at each age.

$$3\;d:\;{\hat{X}}^{\left(1\right)}(c)=-16.63e^{-0.032c}+19.32;$$

$$7\;d:\;{\hat{X}}^{\left(1\right)}(c)=-21.74e^{-0.036c}+25.49;$$

$$28\;d:\;{\hat{X}}^{\left(1\right)}(c)=-33.47e^{-0.026c}+37.85.$$

Generalized solution of the MSC flexural strength model with different levels of GSP doping at each age.

$$3\;d:\;{\hat{X}}^{\left(1\right)}(c)=-8.67e^{-0.062c}+11.16;$$

$$7\;d:\;{\hat{X}}^{\left(1\right)}(c)=-19.47e^{-0.036c}+22.91;$$

$$28\;d:\;{\hat{X}}^{\left(1\right)}(c)=-36.94e^{-0.024c}+41.19.$$

Note: *c* refers to GSP doping.

#### 4.2.2. Model Testing and Analysis

It can be observed from Table 8, Table 9 and Table 10 that the experimental values and GM (1, 1) predicted values are in good agreement, with relative errors for both being within 3.59%. The maximum average error is only 2.14%, indicating that the established GM (1, 1) mechanical strength prediction model for different levels of GSP doping at various ages is a highly precise predictive model capable of accurately estimating MSC mechanical strength values with different levels of GSP doping at each age. Compared with regression analysis and neural network methods, which require a large amount of data and rely on clearly visible patterns of distribution, the GM (1, 1) model demonstrates advantages when applied to testing data that is highly dispersed, has a “small sample,” and is characterized by less information and uncertainty. However, it is important to note that the model is solely applicable to predictions of concrete mechanical strength with GSP concentrations ranging from 0 to 15%. Future work will aim to expand the experimental scheme and optimize the model.

## 5. Conclusions

(1) As GSP doping increases, the characteristic curve of heat of hydration shows a downward trend, while the mechanical strength values of MSC gradually decrease. In the 5% test group, the heat of hydration characteristic curve for the composite cementitious material system was essentially identical to that of the 0% test group, with only small differences observed in the 28-day mechanical strength values between the 5% and 0% test groups of MSC.
(2) During early-stage hydration, GSP acts as a nucleating agent that accelerates the cementitious material system’s hydration process and fills pores. However, excessive dilution of GSP results in fewer C-S-H gel pores in the microstructure, leading to inadequate hydration product formation, a loose structure, and more pores.
(3) Microscopic test results indicate that GSP primarily affects the internal microstructure morphology, pore structure, and interfacial transition zone (ITZ), thereby exerting an impact on the macroscopic mechanical properties of SCM.
(4) The correlation between MSC mechanical strength and pore size distribution parameters, such as gel pore, transition pore, and large pore ash, demonstrates high values, with large pore having the greatest impact on MSC mechanical strength and showing a correlation coefficient above 0.86. The good agreement observed between the predicted and experimental mechanical strength values of MSC using GM (1, 1) models helps in accurately estimating MSC mechanical strength values at each age for different levels of GSP doping.

## Figures and Tables

**Table 1:** Physical properties and chemical composition of raw materials.

Raw Materials	Physical Properties		Chemical Composition (%)						
	Density (kg/m^3^)	Specific Surface Area (m^2^/kg)	SiO_2_	Al_2_O_3_	CaO	Fe_2_O_3_	MgO	Na_2_O	K_2_O
Cement	3070	1890	34.67	7.90	35.35	2.93	1.77	0.75	0.82
GSP	2650	654	63.86	14.92	3.47	2.35	1.02	3.20	3.76
Fly ash	2370	2274	50.77	22.68	5.98	5.64	1.74	1.66	1.80

**Table 2:** Particle size distribution of cement and GSP particles/%.

Raw Materials	Particle Size Distribution				
	<10 μm	10–20 μm	20–40 μm	40–60 μm	>60 μm
Cement	45.98	22.44	22.64	6.85	2.09
GSP	19.20	11.24	19.8	19.44	30.30

**Table 3:** Mixture proportions of concrete (kg/m^3^).

No.	Cement	GSP	Fly Ash	Manufactured Sand	Manufactured Stone	Water	PCA
0%	250	0	100	812.7	1122.3	165	1.75
5%	232.5	17.5					
10%	215	35					
15%	197.5	52.5					

**Table 4:** Experimental scheme for mechanical properties.

No.	Age/d	Test Index
0%	3, 7, 28	Compressive strength, splitting tensile strength, and flexural strength
5%		
10%		
15%		

**Table 5:** Experimental scheme for early-age hydration and microscopic properties.

No.	Test Index
0%	72 h heat of hydration evolution curve, phase composition, SEM images of microstructure characteristics, pore structure, ITZ phase images, ITZ height images, ITZ 3D images, and the roughness of ITZ and slurry
5%	
15%	

**Table 6:** Distribution of MIP pore size in different GSP test groups.

Number	Gel Pore (<10 nm)/%	Transition Pore (10–100 nm)/%	Capillary Pore (100–1000 nm)/%	Large Hole (>1000 nm)/%
0%	5.81	59.61	7.04	27.54
5%	4.93	58.96	12.29	23.83
15%	2.53	60.66	12.69	24.13

**Table 7:** Gray entropy correlation between mechanical strength and pore size distribution parameters.

Pore Size Distribution	<10 nm (Gel Pores)	10–100 nm (Capillary Pores)	100–1000 nm (Transition Pores)	>1000 nm (Large Pores)
Compressive strength	0.846781	0.841416	0.574798	0.861445
Split tensile strength	0.822446	0.865497	0.578182	0.883179
Flexural strength	0.808935	0.875412	0.576903	0.896050

**Table 8:** Test results and calculations of compressive strength GM (1, 1) at different ages.

Age	Content/%	Experimental Value/MPa	Predicted Value/MPa	Residual Error ε/MPa	Relative Error δ/%
3 d	0	25.9	25.9	0	0
	5	22.47	22.10	0.37	1.65
	10	16.43	17.02	0.59	3.59
	15	13.55	13.10	0.45	3.32
7 d	0	35.52	35.52	0	0
	5	32.18	32.12	0.06	0.18
	10	27.15	27.08	0.07	0.25
	15	22.85	22.83	0.02	0.09
28 d	0	46.47	46.47	0	0
	5	42.34	42.29	0.05	0.12
	10	37.89	37.87	0.02	0.05
	15	33.95	33.92	0.03	0.09

**Table 9:** Test results and calculations of splitting tensile strength GM (1, 1) at different ages.

Age	Content/%	Experimental Value/MPa	Predicted Value/MPa	Residual Error ε/MPa	Relative Error δ/%
3 d	0	2.69	2.69	0	0
	5	2.42	2.40	0.02	0.83
	10	2.03	2.06	−0.03	1.48
	15	1.79	1.77	0.02	1.12
7 d	0	3.75	3.75	0	0
	5	3.58	3.59	−0.01	0.28
	10	3.07	3.03	0.04	1.30
	15	2.55	2.57	−0.02	0.78
28 d	0	4.38	4.38	0	0
	5	4.21	4.18	0.03	0.71
	10	3.64	3.69	−0.05	1.37
	15	3.28	3.25	0.03	0.91
	15	3.28	3.25	0.03	0.91

**Table 10:** Test results and calculations of flexural strength GM (1, 1) at different ages.

Age	Content/%	Experimental Value/MPa	Predicted Value/MPa	Residual Error ε/MPa	Relative Error δ/%
3 d	0	2.49	2.49	0	0
	5	2.34	2.33	0.01	0.43
	10	1.74	1.71	0.03	1.72
	15	1.23	1.25	−0.02	1.63
7 d	0	3.44	3.44	0	0
	5	3.30	3.26	0.04	1.21
	10	2.64	2.71	−0.07	2.65
	15	2.31	2.26	0.05	2.16
28 d	0	4.25	4.25	0	0
	5	4.12	4.09	0.03	0.73
	10	3.58	3.64	−0.06	1.68
	15	3.27	3.23	0.04	1.22

## Data Availability

Due to privacy, authors do not agree to release data.
